# Genome-Editing of *FtsZ1* for Alteration of Starch Granule Size in Potato Tubers

**DOI:** 10.3390/plants12091878

**Published:** 2023-05-04

**Authors:** Alexander C. Pfotenhauer, Alessandro Occhialini, Stacee A. Harbison, Li Li, Agnieszka A. Piatek, Curtis R. Luckett, Yongil Yang, C. Neal Stewart, Scott C. Lenaghan

**Affiliations:** 1 Center for Agricultural Synthetic Biology (CASB), University of Tennessee, Knoxville, TN 37996, USA; a.pfotenhauer@utk.edu (A.C.P.); aocchial@utk.edu (A.O.); ssnyde16@utk.edu (S.A.H.); lli88@utk.edu (L.L.); yyang98@utk.edu (Y.Y.);; 2 Department of Plant Sciences, University of Tennessee, Knoxville, TN 37920, USA; curtis@newagemeats.com; 3 Department of Food Science, University of Tennessee, Knoxville, TN 37920, USA; agnieszka.a.piatek@gmail.com

**Keywords:** granule, CRISPR/Cas9, *FtsZ1*, potato, starch

## Abstract

Genome-editing has enabled rapid improvement for staple food crops, such as potato, a key beneficiary of the technology. In potato, starch contained within tubers represents the primary product for use in food and non-food industries. Starch granules are produced in the plastids of tubers with plastid size correlated with the size of starch grana. The division of plastids is controlled by proteins, including the tubulin-like GTPase FtsZ1. The altered expression of *FtsZ1* has been shown to disrupt plastid division, leading to the production of “macro-plastid”-containing plants. These macro-chloroplast plants are characterized by cells containing fewer and enlarged plastids. In this work, we utilize CRISPR/Cas9 to generate *FtsZ1* edited potato lines to demonstrate that genome-editing can be used to increase the size of starch granules in tubers. Altered plastid morphology was comparable to the overexpression of *FtsZ1* in previous work in potato and other crops. Several lines were generated with up to a 1.98-fold increase in starch granule size that was otherwise phenotypically indistinguishable from wild-type plants. Further, starch paste from one of the most promising lines showed a 2.07-fold increase in final viscosity. The advantages of enlarged starch granules and the potential of CRISPR/Cas9-based technologies for food crop improvement are further discussed.

## 1. Introduction

Potato (*Solanum tuberosum*) is the most consumed non-grain food world-wide and has an important position in maintaining global food security for a rapidly expanding population [1,2,3]. Starch is the predominant storage carbohydrate in potato tubers and is utilized in both food and non-food applications [4,5,6,7]. Potato is one of the top sources of industrial starch, and thus the modification of the starch content and composition in potato would significantly impact a variety of industries [8,9,10]. In 2011, the first sequenced potato genome was publicly released, providing key information to enable CRISPR/Cas9 mediated genome-editing for crop improvement [11,12]. CRISPR/Cas9 has demonstrated numerous successes in potato, including increasing amylopectin content, eliminating steroidal glycoalkaloids, and overcoming self-incompatibility [13,14,15,16,17]. Despite the potential of gene editing for agricultural advancements, there is still public concern regarding the safety of genetically modified crops. To address this concern, non-transgenic gene edited potato lines have been engineered through the transient expression of CRISPR/Cas9 [18,19,20]. The expression of CRISPR/Cas9 through plasmid, mRNA, or ribonucleoprotein in protoplasts can allow for gene editing without foreign DNA integration. Engineered potato lines have been successfully regenerated from these genome-edited protoplasts, proving the potential for this pathway for the production of commercial varieties.

The starch produced in the amyloplasts is the most prominent constituent of potatoes [21,22]. Individual amyloplasts in potatoes typically house a single starch granule [23]. Starch comprises amylose and amylopectin stored in the semi-crystalline granules of various sizes. Potato granules tend to be larger than those of cereal starches and granule size is a key parameter for industrial processing. Because of the wide variety of starch applications, there is a desire for granules of differing properties, including increased size [24,25,26,27,28]. Some applications, such as noodle production, favor small granules, though larger granules may increase yield during processing [29,30]. Additionally, it has been shown in sweet potato that smaller starch granules degrade faster than large granules and large granule tubers may be beneficial for storage [31]. Amyloplasts, like all plastids, are organelles that arise from undifferentiated proplastids and through complex signaling differentiate to perform a specific function [32,33,34]. In the case of chloroplasts, the function is photosynthesis, while in amyloplasts it is starch synthesis and storage. Previous studies have demonstrated that plastid division occurs through binary fission with the formation of an electron-dense ring responsible for constriction [35,36,37]. This ring is composed of the Fts (filamentous temperature sensitive) proteins, which are homologous to the proteins responsible for cell division in *Escherichia coli* [38,39,40,41]. The initial discovery of Fts genes in *E. coli* distinguished multiple families, including *FtsZ* [42]. In plants, there are two distinct *FtsZ* gene families, *FtsZ1* and *FtsZ2*, both of which are required for effective plastid division [43]. The expression ratio of the genes is speculated to have an important effect on chloroplast division [44]. Though not fully understood, both increasing and decreasing the expression levels of *FtsZ1* and *FtsZ2* result in altered plastid division, potentially from only one and not both proteins accumulating [45]. The knockdown of either of these in *Arabidopsis* resulted in one or few enlarged chloroplasts, termed “macro-chloroplasts”, as opposed to wild-type cells reported to contain ~100 chloroplasts [36,45]. The authors speculated that a minor change in *FtsZ* levels likely would not affect plastid division and only a severe stoichiometric imbalance of all plastid division components would result in the production of macro-chloroplasts. Regardless of the dramatic change in organelle size and abundance, the phenotypes of resulting plants were similar to wild-type. Specifically, for potato, it has been shown that the decreased expression of *FtsZ1* through RNAi reduces plastid number, while the overexpression of *FtsZ1* results in fewer and larger plastids [46]. Potatoes harvested from macro-plastid lines had larger starch granules and increased phosphate content. Additionally, the viscosity of the starch paste after cooling was higher in macro-plastid lines. In rice endosperm, the knockdown of *FtsZ1* produced larger pleomorphic amyloplasts that initiated division and expanded but did not complete plastid division [47]. Recently, we produced potato lines overexpressing *Arabidopsis thaliana FtsZ1* and proved their capability for chloroplast transformation [48]. These macro-chloroplast lines performed similarly to the wild-type as material for chloroplast transformation but were delayed in growth and yielded less tuber biomass.

Here, CRISPR/Cas9-mediated genome-editing was used for the crop improvement of potato to specifically augment starch granule size by the formation of macro-plastid lines. The use of CRISPR/Cas9 allows for the potential to generate non-transgenic genome-edited potato plants. The transient expression of this system in protoplasts along with plant regeneration could lead to macro-plastid lines similar to the ones described in this work, providing a path to commercialization.

## 2. Results

### 2.1. Generation of Potato Lines with Reduced FtsZ1 Expression Levels

**Figure 1:**
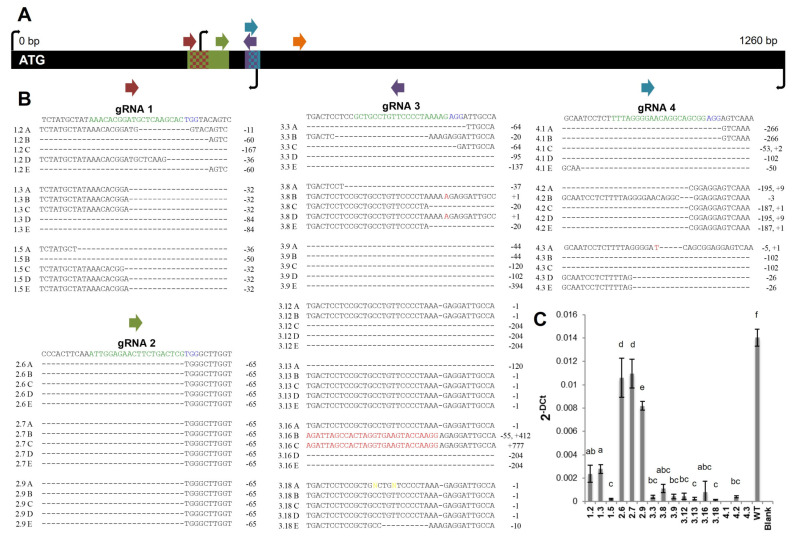
CRISPR/Cas9-mediated genome-editing leads to the reduced expression of potato *FtsZ1*. Several plant lines edited using each gRNA were selected for sequencing (**A**). The sequence displayed under each gRNA corresponds to WT sequence. The green and blue highlighted sequence corresponds to that of the gRNA and PAM, respectively. The characterization of each mutation is annotated on the right of each sequence. Schematic of gRNAs, deleted DNA regions, and primers on *FtsZ1* coding sequence (**B**). The entire black bar is representative of the 1260 bp coding sequence of *FtsZ1*. Colored arrows represent gRNAs. Colored rectangles on the *FtsZ1* black bar represent the deleted regions of DNA, checkerboard rectangles represent areas deleted in multiple lines. Black arrows represent primers used for RT and qRT-PCR. qRT-PCR was conducted on *FtsZ1* vs. EF1 (**C**). Results are expressed as 2^−ΔCt^ mean ± standard deviation of the three experiments. Means were compared with ANOVA, followed by Tukey–Kramer post hoc analysis using a *p*-value of 0.05. Different letters above plotted means indicate a statistically significant difference. WT = wild-type. gRNA = guide RNA. Bp = base pair. PAM = protospacer adjacent motif.

*FtsZ1* was chosen over *FtsZ2* for gene editing, as *FtsZ1* repression alone has been shown to affect plastid division and *FtsZ2* has not been accurately identified in potato. The entire *FtsZ1* coding sequence was cloned and sequenced from *S. tuberosum* var. ‘Desirée’ cDNA to accurately design gRNAs. gRNAs were designed on exonic DNA. Following this, fifty-six transgenic lines from five different gRNA constructs were produced from transformation with *Agrobacterium tumefaciens*. Six transgenic lines were produced with gRNA1, twelve with gRNA2, eighteen with gRNA3, eight with gRNA4, and twelve with gRNA5. A restriction digest assay was performed to identify mutant lines (Supplementary Figure S1). Six plants were mutated from gRNA1 (100%), eleven from gRNA2 (92%), thirteen from gRNA3 (72%), eight from gRNA4 (100%), and zero from gRNA5 (0%) (thirty-eight in total). No lines were generated with only undigested bands, which would be indicative of one homozygous mutation carried through all four alleles. Three or more lines from each gRNA were chosen for sequencing to characterize the type of mutagenesis caused by Cas9 cleavage and all 16 lines had mutations (Figure 1A,B). Five clones of *FtsZ1* cDNA are shown and represent some of the mutations found in these lines. Lines produced with gRNA1 had deletions ranging from 11 to 167 base pairs. Every line produced with gRNA2 had a −65 base pair deletion. Lines produced with gRNA3 had deletions and insertions ranging from −/+1 to −394 or +777 base pairs. Interestingly, the +412 and +777 fragments inserted in line 3.16 aligned with 100% homology to an unannotated mitochondrial DNA sequence (Supplementary Table S1). This insert appears to be a non-coding sequence. Lines produced with gRNA4 had deletions ranging from −3 to −266 base pairs but only a maximum of +9 base pair insertion. No lines that had non-frameshift mutations, such as indels of three or six base pairs, were continued in this study, lending confidence that the genome-edited lines should influence FtsZ1 function, given that coding sequence ought to result in impaired transcripts and subsequent peptide sequence. Indels within intron-spanning sequence would be expected to cause disruptions in transcription and affect FtsZ1 synthesis. The PCR amplification of genomic DNA *FtsZ1* fragments for the 16 down-selected lines were congruent with sequencing results and displayed large genomic deletions and insertions (Supplementary Figure S2). All 16 lines showed multiple amplification patterns, indicating that none of the lines were homozygous mutants.

cDNA was produced from the 16 lines and analyzed using RT-PCR and qRT-PCR. Full-length 1260 base pair (bp) *FtsZ1* amplification analysis indicated that many lines had multiple band amplifications or very low amplification (Supplementary Figure S3). Lower than wild-type molecular weight amplicons corroborate sequencing data that indicate large portions of *FtsZ1* were deleted following DNA repair. Primers for qRT-PCR were designed on conserved deleted regions among all 16 lines (Figure 1B). qRT-PCR analysis showed that all lines had a decrease in *FtsZ1* expression compared to wild-type, ranging from a 1.3- to 115-fold reduction (Figure 1C). Lines 2.6, 2.7, and 2.9 expressed *FtsZ1* less than wild-type but more than other transgenic lines, likely due to fewer alleles being targeted by Cas9. Potentially, lines 2.6, 2.7, and 2.9 still contain one or more wild-type *FtsZ1* allele. Lines 4.1 and 4.3 did not produce a detectable transcript using the primers for qRT-PCR. The decreased qRT-PCR amplification of *FtsZ1* in mutant lines is likely due to sequence complementary to either primer 13 or 14 being destroyed.

### 2.2. The Reduction in FtsZ1 Expression Produced Potato Tubers with Increased Starch Granule Size without Comprising Nutritional Quality

**Figure 2:**
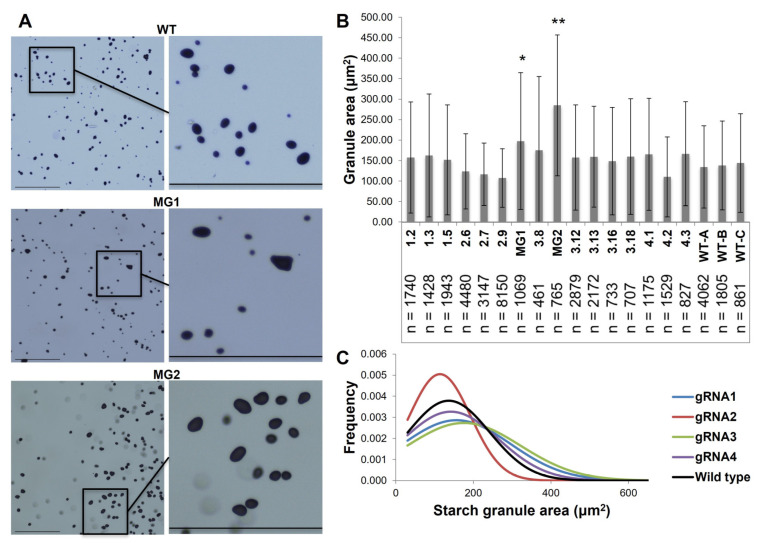
Reduced *FtsZ1* expression leads to the production of larger starch granules. Microtuber starch granules were analyzed via light microscopy (**A**). Tubers were scraped onto slides and stained. Scale bar = 275 μm. Granule area (μm^2^) mean ± standard deviation (**B**). The number of granules analyzed for each microtuber is noted by n. Means were compared with ANOVA, followed by Tukey–Kramer post hoc analysis (* = *p* < 0.05, ** = *p* < 0.01 different from all other lines). The distribution of the granule area for compiled lines from each gRNA (**C**). WT = wild-type. MG1 = MacroGranule1. MG2 = MacroGranule2. gRNA = guide RNA.

Microtubers were generated from all 16 lines. Starch granule size from these microtubers was determined by light microscopy (Figure 2A). Since there is a direct relationship between tuber size and starch granule size, only microtubers ranging from 10 to 30 mg were used (Supplementary Table S2) [49]. Three wild-type microtuber controls between 13.6 and 29.7 mg were included and none were significantly different from one another. Lines 3.3 and 3.9 contained starch granules significantly larger than wild-type (1.37- and 1.98-fold larger, respectively) or any other transgenic lines (Figure 2B). Based on this criteria, lines 3.3 and 3.9 were pursued for further analysis and will be referred to as MacroGranule1 and MacroGranule2 from this point forward. Leaf tissue from the 16 downselected mutants and wild-types was analyzed using confocal microscopy (Supplementary Figure S4). Several lines (MG1, MG2, 3.12, and 4.2) contain fewer and larger chloroplasts compared to the wild-type. The morphology of the chloroplasts, especially for MacroGranule2, was less uniform than the typical ovoid shape observed in the wild-type.

All 16 lines were grown in a greenhouse to produce tubers for subsequent analysis (Supplementary Figure S5). Tubers were analyzed using a variety of methods to determine total phosphorus, phosphate, fat, nitrogen, protein, starch, and amylose content (Table 1). All lines, apart from lines 4.1 and 4.3, contained the same nutritional content as the wild-type. Lines 4.1 and 4.3 had a significant decrease in starch content. Lines were also compiled by gRNA and compared to wild-type, and no compiled gRNA groups were significantly different from each other (Table 1). These results indicated that there was no nutritional penalty for the investigated macro and micronutrients following the genome-editing of FtsZ1.

### 2.3. The Phenotypes and Growth Characteristics of MacroGranule1 and MacroGranule2 Plants Were Comparable to Non-Edited Wild-Type Plants

**Figure 3:**
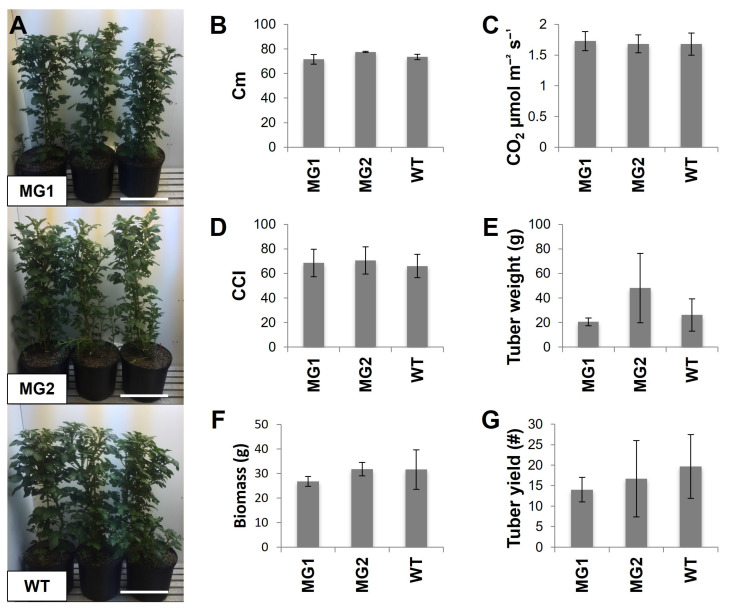
Phenotypic analysis of macro-plastid lines. Photos of each line growing in an 11.4 l pot shortly before bolting (**A**). Scale bar = 30 cm. Plant height in centimeters (Cm) (**B**). Leaf CO_2_ assimilation (**C**). Chlorophyll concentration index (CCI) (**D**). Total tuber weight (**E**). Biomass of above ground tissue dry weight (**F**). Total tuber yield (**G**). Results are expressed as mean ± standard deviation. Means were compared with ANOVA, followed by Tukey–Kramer post hoc analysis. WT = wild-type. MG1 = MacroGranule1. MG2 = MacroGranule2. # = number of tubers.

MacroGranule1, MacroGranule2, and wild-type plants were chosen for a second growth study to analyze additional phenotypic parameters (Figure 3). Both transgenic lines reached the same height, contained the same level of chlorophyll, and conducted gas exchange congruent to the wild-type. No differences in tuber yield or aboveground biomass were observed (Supplementary Figure S6). In contrast to our previous overexpression of FtsZ1 potato lines [48], MacroGranule1 and MacroGranule2 plants did not display any delay in growth. These results lend confidence that *FtsZ1* mutants can be successfully grown to produce macro-plastid potatoes.

### 2.4. MacroGranule2 Starch Displays a Higher Viscosity Level than MacroGranule1 and Wild-Type

**Figure 4:**
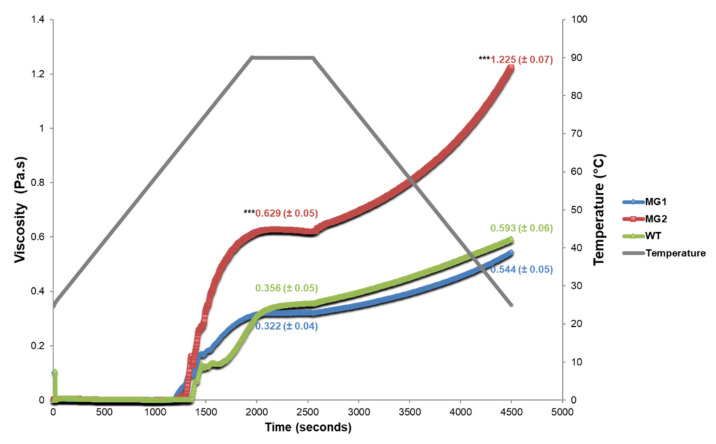
MacroGranule2 starch granules show increased viscosity compared to MacroGranule1 and wild-type. Values indicate maximum viscosity during the 90 °C holding phase or final viscosity, and colors are maintained for the three lines. Results are expressed as mean ± standard deviation. Means were compared with ANOVA, followed by Tukey–Kramer post hoc analysis (*** = *p* < 0.001 different from WT). WT = wild-type. MG1 = MacroGranule1. MG2 = MacroGranule2.

The starch isolated from tubers of MacroGranule1, MacroGranule2, and wild-type lines was used to determine pasting properties through the use of a rheometer. The viscosity of all three lines began increasing at around 65 °C (Figure 4). MacroGranule1 and wild-type reached a maximum viscosity during the 90 °C hold phase of 0.322 (±0.04) and 0.356 (±0.05) Pa.s, respectively. Interestingly, MacroGranule2 reached a maximum viscosity during the hold phase of 0.629 (±0.05) Pa.s, a 1.77-fold increase over wild-type. MacroGranule1 and wild-type reached a final viscosity of 0.544 (±0.05) and 0.593 (±0.06) Pa.s, respectively. MacroGranule2 reached a final viscosity of 1.225 (±0.07) Pa.s, a 2.07-fold increase over wild-type.

## 3. Discussion

We show here that a reduction in *FtsZ1* expression mediated by CRISPR/Cas9 can produce macroplastid lines that contain larger starch granules (Figure 2 and Supplementary Figure S3). Though only one gRNA was used to generate each mutant line, large insertions and deletions were frequently detected. These large mutations have been previously reported in plants, including in potato [50,51,52]. Despite constitutive Cas9 expression, no lines had homozygous mutations, as determined by a restriction digest assay and the simple PCR of genomic DNA (Supplementary Figures S1 and S2). This may be due to allelic differences in *FtsZ1*, especially considering the tetraploid genome structure of potato. Several of the lines potentially arose from the same piece of transformed callus. Lines 3.12, 3.13, and 3.16 all had the same distinct amplification pattern and are likely genetically indistinguishable (Supplementary Figure S2). Several large amplicon insertions detected through sequencing of line 3.16 could also potentially be detected in lines 3.12 and 3.13 with additional technical replicates. Targeted deep amplicon sequencing was attempted for all lines but was unsuccessful due to the tetraploid genome of potato.

The large 412 and 777 base pair insertion detected in line 3.16 is particularly interesting. These sequences align with 100% homology to unannotated potato mitochondrial DNA. These large insertions also align with high homology to other Solanum species’ mitochondrial DNA, including tomato and eggplant. Mitochondrial DNA has been previously reported to insert into nuclear genomes in eukaryotes, including plants with up to a 620 kilobase insertion in *A. thaliana* [53,54,55]. Mitochondrial DNA insertion is thought to occur following double-strand breaks, and to the best of our knowledge, this is the first recorded instance mediated through cleavage via CRISPR/Cas9 or any other genome-editor [56].

Though most were not significant, 75% of the regenerated mutants produced starch granules were at least slightly larger than wild-type (Figure 2). Two lines, MacroGranule1 and MacroGranule2, produced starch granules 1.37- and 1.98-fold larger than wild-type potato, respectively. MacroGranule1 and MacroGranule2 were generated using gRNA3, and all seven lines from gRNA3 created microtubers with at least slightly larger starch granules compared to wild-type (Figure 2 and Supplementary Table S2). gRNA3 directs Cas9 to a putative cut site at 20 bp 3′ of an intron splice site. This close proximity to the splice site may explain a higher effect seen in these lines. Potentially, the mutations in MacroGranule1 and MacroGranule2 resulted in a more deleterious FtsZ1 protein that aberrantly affected the Z-ring formation more than FtsZ1 proteins in other mutant lines. The macro-plastid phenotype was also most dramatic in MacroGranule1 and MacroGranule2 compared to other mutants, which likely led to the formation of the larger starch granules (Supplementary Figure S4). Although several lines other than MacroGranule1 and MacroGranule2 showed altered chloroplast morphology, this did not translate to statistically different starch granule sizes. This may be due to lower overall *FtsZ1* expression in tubers compared to leaves and, ultimately, a lessened effect in the tuber plastids.

Previous work using RNAi demonstrated that a reduction in *FtsZ1* can affect plastid morphology and plastid division patterns; however, this work demonstrated that a more viable commercial approach, through CRISPR/Cas9 mediated genome-editing, can result in a similar larger starch granule phenotype [46,47]. Our previous generation of macro-plastid lines generated through the overexpression of *A. thaliana FtsZ1* produced lines that were delayed in growth and had lower tuber biomass [48]. In this study, MacroGranule1 and MacroGranule2 produced the same tuber biomass as the wild-type without a delay in growth (Figure 3). Interestingly, there was no increase in phosphate content as was found previously [46]. The lack of delayed growth patterns observed in these lines may be due to differences in the expression levels of *FtsZ1* as compared to previous overexpressing lines [57]. The overexpression of *FtsZ1* and its effect on chloroplast division has shown to be dose-dependent, and perhaps a moderate decrease in expression could result in plants with intermediate-sized plastids [45]. MacroGranule1 and MacroGranule2 may fall into this category of macro-plastid plants, retaining adequate growth patterns while still producing larger starch granules. Previous research has shown that suspensions of larger starch granules exhibit increased viscosity characteristics [58,59,60]. MacroGranule2 contained starch granules almost twice as large as wild-type, and this likely led to the increased final viscosity (Figure 4). Potentially, the 1.37-fold increase in MacroGranule1 starch granule size is not enough to affect final viscosity. As MacroGranule2′s starch paste reached a maximum viscosity twice as high as that of the wild-type, it could potentially be used in smaller amounts. Additionally, blends of different starch sources are commonly used to fit a specific purpose, and tubers from MacroGranule2 may be useful to fit a distinct need [61,62,63]. Potato starch paste has a very high level of clarity, and this benefit may increase the usefulness of starch from MacroGranule2 [64].

While CRISPR/Cas9 has revolutionized plant biotechnology, there is still public skepticism regarding genetically engineered crops and a significant hurdle to bringing transgenic crops to market [65,66,67]. In the current regulatory landscape in the U.S., an attractive alternative is to produce plants through transient genome-editing, using preassembled ribonucleoproteins or mRNA [68,69,70]. The delivery of CRISPR/Cas9 cargo through either of these methods using protoplasts or biolistics ensures that DNA editing can occur without the possibility of foreign DNA integration. These methods could potentially be applied with our gRNA to generate lines similar to MacroGranule1 and MacroGranule2 for the commercial production of a large starch granule potato line.

Here, we have described the generation of larger starch granule potato tubers using CRISPR/Cas9-mediated genome-editing. The two plant lines produced, MacroGranule1 and MacroGranule2, grew without a fitness penalty and produced tubers with larger starch granules but otherwise similar phenotypes and nutritional profiles. Our design could be taken further via a DNA-free genome-editing platform to produce larger starch granule potatoes suitable for the modern market.

## 4. Materials and Methods

### 4.1. FtsZ1 CDS Cloning/Sequencing

*S. tuberosum* var. ‘Desirée’ were grown in Magenta GA7 boxes with MS Reg media [48]. RNA was extracted from ~1-month-old tissue using TRI Reagent, as per the manufacturer’s instructions (Molecular Research Center, Cincinnati, OH, USA). RNA was cleaned with a Zymo Research RNA Clean and Concentrator kit (Irvine, CA, USA). cDNA was synthesized according to protocol using a Thermo Fisher Scientific SuperScript III First-Strand Synthesis System (Waltham, MA, USA). The *FtsZ1* coding sequence was amplified using primers 1 and 2 (all primers used in this study can be found in Supplementary Table S3). The *FtsZ1* amplicon and pUC19 were digested with XbaI and HindIII (NEB, Ipswich, MA, USA). One microliter of calf intestinal alkaline phosphatase was added to the pUC19 digestion for dephosphorylation, and the amplicon was ligated into the vector. The vector was Sanger sequenced with M13 forward and reverse primers.

### 4.2. Vector Construction

pKSE401 was obtained through Addgene (plasmid number 62202) (Watertown, MA, USA) [71]. The vector was modified to have hygromycin plant selection, as opposed to kanamycin selection. Briefly, a hygromycin phosphotransferase-encoding gene from plasmid pMDC32 was amplified using primers 3 and 4 [72]. The hygromycin amplicon and pKSE401 were digested with NcoI and SacII. One microliter of calf intestinal phosphatase was added to the pKSE401 digestion, and the hygromycin fragment was ligated into the vector, now named pKSE401-Hyg. Five guide RNAs (gRNAs) were designed on two different *FtsZ1* exons by considering GC content, unique restriction enzyme recognition sites, and off-target effects by using CRISPOR (Supplementary Table S3) [73]. The off-target effect was minimal due to the use of CRISPOR, and therefore, gRNAs should not target *FtsZ2* or any potentially similar sequences. gRNAs were designed to direct Cas9 to a restriction enzyme recognition site. Upon Cas9 cleavage and subsequent DNA repair, indels destroyed these recognition sites. Undigested PCR fragments were thus indicative of mutagenesis. gRNAs were designed with BsaI overhangs and ordered as complementary single stranded oligonucleotides (IDT, Coralville, IA, USA). gRNAs were annealed at 95 °C for two minutes in oligo annealing buffer (10 mM Tris HCl, pH 8.0; 50 mM NaCl; 1 mM EDTA) and cloned into the pKSE401-Hyg vector using BsaI sites.

### 4.3. Plant Transformation

pKSE401-Hyg constructs were transformed into *A. tumefaciens* strain LBA4404 using the freeze–thaw method [74]. One-month old in vitro potato cultures were used for plant transformation as previously described [75]. Briefly, *A. tumefaciens* was grown for several hours to reach an optical density of 0.7 in liquid YEP media with rifampicin and kanamycin at 50 mg/L. Cultures were spun down and resuspended in liquid CIM media at an optical density of 0.7 [75]. Potato internodes of 1 cm in size were plated onto solid CIM plates, and explants were inoculated with the *A. tumefaciens* culture for 20 min [75]. Internodes were moved to new CIM plates and placed in the dark for 3 days before being moved to 3C5ZR plates supplemented with 20 mg/mL hygromycin [75]. Plantlets were transferred to new 3C5ZR plates every 10 days until shoots emerged, when they were then transferred to MS Reg media supplemented with 20 mg/mL hygromycin [48]. Fifty-six putative transgenic plantlets were chosen for genomic DNA extraction based on their ability to form roots in media containing hygromycin.

### 4.4. Genomic DNA Extraction and Molecular Analysis

DNA was extracted from the 56 putative transgenic plantlets with the CTAB method [76]. PCR was performed with primers 5 and 6 on 20 ng genomic DNA to check for the presence of Cas9 DNA. All 56 lines were then subjected to the restriction enzyme digest assay. Twenty nanograms of genomic DNA from each of the plantlets generated with gRNAs 1 and 2 were amplified with primers 7 and 8; while plantlets generated with gRNAs 3, 4, and 5 were amplified with primers 9 and 10. Following amplification, the PCR products were purified, diluted to 900 ng, and digested with appropriate restriction enzymes overnight (Supplementary Table S3). The digestions were run on an agarose gel and checked for undigested bands. All undigested bands were cut out and cleaned with a Qiagen QIAquick Gel Extraction Kit (Hilden, Germany).

### 4.5. Cloning and Sequencing of Mutated Amplicons

pUC19 was digested with 1 μL HincII and 1 μL calf intestinal phosphatase. The undigested DNA bands from 16 lines were re-amplified using the same phosphorylated primers and ligated into the vector. Following colony PCR, 5 putative positive colonies (A-E) per transgenic line were used for plasmid isolation and were Sanger sequenced with M13 forward and reverse primers.

### 4.6. RT PCR and q-RT PCR

cDNA was synthesized as before for all 16 down-selected lines. RT-PCR was conducted using primers 11 and 12 to amplify the entire 1260 bp *FtsZ1* coding sequence and 15 and 16 to amplify a portion of *EF1α* as a control. Primers 13 and 14 and 15 and 16 were used to amplify a portion of *FtsZ1* or *EF1α*, respectively, for qRT-PCR. Primers 13 and 14 were designed on a 97 bp portion of *FtsZ1*, which was removed following CRISPR/Cas9 mutagenesis in all lines. Therefore, the amplification of cDNA would not occur on deleted sequence due to the lack of a template. Because all regenerated lines are heterozygous, some native *FtsZ1* expression can still be detected in transgenic lines and compared to wild-type. qRT-PCR reactions were performed using the PowerUp SYBR Green Master Mix (Thermo Scientific, Waltham, MA, USA) and run on a QuantStudio 6 Flex. The data are expressed as 2^−ΔCT^ of *FtsZ1* vs. *EF1α*. Results are shown as mean ± standard deviation of 3 technical replicates.

### 4.7. Starch Granule Size Determination

Plantlets were grown on plates containing microtuber induction media: 2.89 g/L Murashige-Skoog Salts (without vitamins); 1.04 g/L B5 salts (Gamborg B5 basal); 5 mL “complete” vitamin stock solution (400 g/L glycine; 100 mg/L nicotinic acid; 100 mg/L pyridoxine HCl; 100 mg/L thiamine HCl); 330 μg/L folic acid; 500 μg/L d-biotin; 10 mg/L kinetin; 100 μg/L indole acetic acid; 100 mg/L inositol; 80 g/L sucrose; 6 g/L agar; and pH 5.7. One centimeter internode segments, with leaves removed, were placed on media that was kept in the dark at 17 °C. After one month of growth, microtubers were transferred onto microscope slides, and stained with a 1:1 mixture of Lugol’s solution (6.7 g/L potassium iodide; 3.3 g/L iodine) to glycerol. Light microscope images were used to assess the size of the stained starch granules. Granule area was measured using ImageJ 1.41 from the National Institute of Health (Supplementary Table S2) (Bethesda, ML, USA).

### 4.8. Confocal Microscopy

One-month-old fully expanded leaves were analyzed using an Olympus Fv1000 confocal microscope (Tokyo, Japan). Chlorophyll autofluorescence was excited using a 543 nm helium–neon laser and was detected with an emission wavelength of 667 nm. Images were taken with the same parameters using Olympus FV10-ASW Viewer software Ver.4.2a. The images were processed with the ImageJ 1.41o software from the National Institute of Health.

### 4.9. Growth Studies

In vitro potato tissue that was 1 month old was propagated from the 16 genome-edited lines and wild-type control in MS Reg media [48]. After roots emerged ~2 weeks, 5 apical meristems from each line and wild-type were transferred to Pro-Mix BK25 potting mix and grown under fluorescent lights at ambient temperature in 6 × 6 × 9 cm pots. Six weeks later, the plants were transferred to 11.4 l pots and grew until senescence in a greenhouse. Tubers were collected and washed with deionized water.

The triplicate replicates of MacroGranule1, MacroGranule2, and wild-type plants were propagated and transferred to potting mix, as described previously. Plants were grown in growth chambers with 16/8 h of light/dark cycle and a constant temperature of 22 °C. Phenotypic analysis was conducted simultaneously for all lines as flowers began to emerge. CO_2_ assimilation values per unit of leaf area (μmol m^−2^ s^−2^) were measured with a LI-COR Biosciences LI-6800 Portable Photosynthesis System (Lincoln, NE, USA) with atmospheric CO_2_ conditions (400 μmol mol air^−1^), constant irradiance (1000 μmol photons m^−2^ s^−2^), 23 °C, a vapor pressure deficit of 0.8–1.2 kPa, and a flow rate of 200 μmol s^−1^. An Opti-Sciences CCM-200plus chlorophyll meter (Hudson, NH, USA) was used to measure chlorophyll content index (CCI). Three readings from three different leaves (nine in total) were taken from each plant. Tubers were collected, cleaned with deionized water, dried at ambient temperature, and weighed. All above-ground tissue was collected, dried at 50 °C, and weighed.

### 4.10. Tuber Analysis

Tubers from the 16 genome-edited lines and wild-type were sent to Eurofins Food Testing Services (Des Moines, IA, USA) for the determination of total fat, phosphorus, phosphate, nitrogen, and protein content. The association of Official Agricultural Chemists methods 990.03, 992.15, 954.02, modified 984.27, modified 927.02, modified 985.01, and modified 965.17 were conducted. Single values were provided for individual lines and compiled among similar gRNA groups, which are shown as mean ± standard deviation. Tubers were also lyophilized and milled to determine total starch and amylose content using Megazyme kits K-TSTA-100A and K-AMYL (Wicklow, Ireland).

### 4.11. Starch Pasting Properties

Starch was extracted from MacroGranule1, MacroGranule2, and wild-type tubers as previously described [77]. Pasting properties were analyzed with previously described methods on a Discovery HR-2 Rheometer using a 40 mm Peltier steel parallel plate (New Castle, DE, USA) [78].

### 4.12. Statistical Analysis

Means were compared via ANOVA, followed by Tukey–Kramer (*p* < 0.05 = *, *p* < 0.01 = **, *p* < 0.001 = ***) using CoStat software (Cohort Software Ltd., Birmingham, UK).

## Figures and Tables

**Table 1:** Nutritional analysis of *FtsZ1* edited tubers. Tubers from greenhouse-grown plants were analyzed for the determination of total phosphorus, phosphate, fat, nitrogen, protein, starch, and amylose. Single values are shown for individual lines for phosphorus, phosphate, fat, nitrogen, and protein. Results for starch and amylose for individual lines, and all parameters for compiled gRNAs are expressed as mean ± standard deviation. Means were compared by ANOVA, followed by Tukey–Kramer (** = *p* < 0.01 different from all other lines). MG1 = MacroGranule1. MG2 = MacroGranule2. WT = wild-type. gRNA = guide RNA.

Line/s	Phosphorus %	Phosphate %	Fat %	Nitrogen %	Protein %	Starch %	Amylose %
1.2	0.11	0.34	0.36	0.61	3.81	59.05 (±0.89)	29.86 (±0.50)
1.3	0.11	0.34	0.15	0.61	3.81	55.44 (±1.22)	30.28 (±0.32)
1.5	0.10	0.31	0.32	0.50	3.13	59.82 (±1.74)	29.99 (±1.18)
2.6	0.11	0.33	0.10	0.50	3.13	57.79 (±2.38)	29.10 (±0.47)
2.7	0.12	0.35	0.16	0.58	3.63	57.63 (±1.96)	25.58 (±0.265)
2.9	0.12	0.36	0.18	0.54	3.38	58.95 (±1.12)	27.64 (±0.58)
MG1	0.12	0.36	0.27	0.55	3.44	55.80 (±1.07)	27.10 (±0.50)
3.8	0.12	0.38	0.15	0.64	4.00	58.75 (± 0.41)	28.89 (± 0.53)
MG2	0.13	0.41	0.10	0.61	3.81	57.83 (±0.63)	26.16 (±0.41)
3.12	0.11	0.34	0.15	0.55	3.44	56.89 (±0.83)	27.04 (±0.42)
3.13	0.11	0.35	0.10	0.56	3.50	56.51 (±0.37)	30.04 (±0.56)
3.16	0.11	0.33	0.26	0.54	3.38	58.76 (±0.45)	29.12 (±0.45)
3.18	0.11	0.33	0.30	0.53	3.31	57.69 (±0.73)	29.09 (±0.40)
4.1	0.11	0.33	0.26	0.58	3.63	** 50.39 (±0.84)	27.63 (±0.38)
4.2	0.12	0.38	0.17	0.71	4.44	-	-
4.3	0.13	0.39	0.29	0.73	4.56	** 48.74 (±0.37)	28.33 (±0.58)
WT	0.12	0.37	0.19	0.67	4.19	54.14 (±0.54)	28.70 (±0.56)
Compiled gRNA1	0.11 (±0.01)	0.33 (±0.02)	0.28 (±0.11)	0.57 (±0.06)	3.58 (±0.39)	58.10 (±2.34)	30.04 (±0.21)
Compiled gRNA2	0.11 (±0.01)	0.35 (±0.01)	0.15 (±0.04)	0.54 (±0.04)	3.38 (±0.25)	58.12 (±0.72)	27.44 (±1.77)
Compiled gRNA3	0.12 (±0.01)	0.36 (±0.03)	0.19 (±0.08)	0.57 (±0.04)	3.55 (±0.25)	57.46 (±1.12)	28.21 (±1.43)
Compiled gRNA4	0.12 (±0.01)	0.37 (±0.03)	0.27 (±0.02)	0.67 (±0.08)	4.21 (±0.51)	49.56 (±1.17)	27.98 (±0.49)

## Data Availability

Raw data presented in this study are available on request from the corresponding author.

## Supplementary Materials

The following supporting information can be downloaded at: https://www.mdpi.com/article/10.3390/plants12091878/s1, Supplementary Table S1: Mitochondrial DNA insertion in line 3.16; Supplementary Table S2: Microtuber weight and mean area; Supplementary Table S3: Primers and gRNAs; Supplementary Figure S1: Restriction digest assay; Supplementary Figure S2: PCR amplification of FtsZ1 fragments; Supplementary Figure S3: FtsZ1 expression patterns; Supplementary Figure S4: Chloroplast morphology by confocal microscopy of large starch granule lines; Supplementary Figure S5: Greenhouse production of FtsZ1 mutant potato lines; Supplementary Figure S6: Tubers produced from MacroGranule1, MacroGranule2, and WT.

## References

[1] Barrell P.J., Meiyalaghan S., Jacobs J.M., Conner A.J. (2013). Applications of biotechnology and genomics in potato improvement. Plant Biotechnol. J..

[2] Lutaladio N.C., Castaldi L. (2009). Potato: The hidden treasure. J. Food Compos. Anal..

[3] Devaux A., Kromann P., Ortiz O. (2014). Potatoes for sustainable global food security. Potato Res..

[4] Chakraborty R., Kalita P., Sen S. (2019). Natural starch in biomedical and food Industry: Perception and overview. Curr. Drug Discov. Technol..

[5] Ellis R.P., Cochrane M.P., Dale M.F.B., Duffus C.M., Lynn A., Morrison I.M., Prentice R.D.M., Swanston J.S., Tiller S.A. (1999). Starch production and industrial use. J. Sci. Food Agric..

[6] Kraak A. (1992). Industrial applications of potato starch products. Ind. Crops Prod..

[7] Burrell M.M. (2003). Starch: The need for improved quality or quantity—An overview. J. Exp. Bot..

[8] Jobling S. (2004). Improving starch for food and industrial applications. Curr. Opin. Plant Biol..

[9] Tharanathan R.N. (2005). Starch—Value addition by modification. Crit. Rev. Food Sci. Nutr..

[10] Davis J.P., Supatcharee N., Khandelwal R.L., Chibbar R.N. (2003). Synthesis of novel starches *in planta*: Opportunities and challenges. Starch.

[11] Xu X., Pan S., Cheng S., Zhang B., Mu D., Ni P., Zhang G., Yang S., Li R., Wang J. (2011). Genome sequence and analysis of the tuber crop potato. Nature.

[12] Cong L., Ran F.A., Cox D., Lin S., Barretto R., Habib N., Hsu P.D., Wu X., Jiang W., Marraffini L.A. (2013). Multiplex genome engineering using CRISPR/Cas systems. Science.

[13] Butler N.M., Atkins P.A., Voytas D.F., Douches D.S. (2015). Generation and inheritance of targeted mutations in potato (*Solanum tuberosum* L.) using the CRISPR/Cas system. PLoS ONE.

[14] Wang S., Zhang S., Wang W., Xiong X., Meng F., Cui X. (2015). Efficient targeted mutagenesis in potato by the CRISPR/Cas9 system. Plant Cell Rep..

[15] Andersson M., Turesson H., Nicolia A., Fält A.S., Samuelsson M., Hofvander P. (2017). Efficient targeted multiallelic mutagenesis in tetraploid potato (*Solanum tuberosum*) by transient CRISPR-Cas9 expression in protoplasts. Plant Cell Rep..

[16] Nakayasu M., Akiyama R., Lee H.J., Osakabe K., Osakabe Y., Watanabe B., Sugimoto Y., Umemoto N., Saito K., Muranaka T. (2018). Generation of α-solanine-free hairy roots of potato by CRISPR/Cas9 mediated genome editing of the St16DOX gene. Plant Physiol. Biochem..

[17] Enciso-Rodriguez F., Manrique-Carpintero N.C., Nadakuduti S.S., Buell C.R., Zarka D., Douches D. (2019). Overcoming self-incompatibility in diploid potato using CRISPR-Cas9. Front. Plant Sci..

[18] Veillet F., Perrot L., Chauvin L., Kermarrec M.P., Guyon-Debast A., Chauvin J.E., Nogue F., Mazier M. (2019). Transgene-free genome editing in tomato and potato plants using *Agrobacterium*-mediated delivery of a CRISPR/Cas9 cytidine base editor. Int. J. Mol. Sci..

[19] Johansen I.E., Liu Y., Jørgensen B., Bennett E.P., Andreasson E., Nielsen K.L., Blennow A., Petersen B.L. (2019). High efficacy full allelic CRISPR/Cas9 gene editing in tetraploid potato. Sci. Rep..

[20] Tuncel A., Corbin K.R., Ahn-Jarvis J., Harris S., Hawkins E., Smedley M.A., Harwood W., Warren F.J., Patron N.J., Smith A.M. (2019). Cas9-mediated mutagenesis of potato starch-branching enzymes generates a range of tuber starch phenotypes. Plant Biotechnol. J..

[21] Naeem M., Tetlow I.J., Emes M.J. (2002). Starch synthesis in amyloplasts purified from developing potato tubers. Plant J..

[22] Smith A.M., Denyer K., Martin C. (1997). The synthesis of the starch granule. Annu. Rev. Plant Physiol. Plant Mol. Biol..

[23] Kram A.M., Oostergetel G.T., Van Bruggen E. (1993). Localization of branching enzyme in potato tuber cells with the use of immunoelectron microscopy. Plant Physiol..

[24] Ji Q., Oomen R.J., Vincken J.P., Bolam D.N., Gilbert H.J., Suurs L.C., Visser R.G. (2004). Reduction of starch granule size by expression of an engineered tandem starch-binding domain in potato plants. Plant Biotechnol. J..

[25] Lindeboom N., Chang P.R., Tyler R.T. (2004). Analytical, biochemical and physicochemical aspects of starch granule size, with emphasis on small granule starches: A review. Starch.

[26] Park S.H., Wilson J.D., Seabourn B.W. (2009). Starch granule size distribution of hard red winter and hard red spring wheat: Its effects on mixing and breadmaking quality. J. Cereal Sci..

[27] Smith A.M., Martin C. (1993). Biosynthesis and Manipulation of Plant Products.

[28] Stoddard F.L. (2003). Genetics of starch granule size distribution in tetraploid and hexaploid wheats. Aust. J. Agric. Res..

[29] Chen Z., Schols H.A., Voragen A.G.J. (2006). Starch granule size strongly determines starch noodle processing and noodle quality. J. Food Sci..

[30] Gutierrez O.A., Campbell M.R., Glover D.V. (2002). Starch particle volume in single- and double-mutant maize endosperm genotypes involving the soft starch (h) gene. Crop. Sci..

[31] Niu S., Li X.Q., Tang R., Zhang G., Li X., Cui B., Mikitzel L., Haroon M. (2019). Starch granule sizes and degradation in sweet potatoes during storage. Postharvest Biol. Technol..

[32] Liebers M., Grübler B., Chevalier F., Lerbs-Mache S., Merendino L., Blanvillain R., Pfannschmidt T. (2017). Regulatory shifts in plastid transcription play a key role in morphological conversions of plastids during plant development. Front. Plant Sci..

[33] Pyke K.A. (1999). Plastid division and development. Plant Cell.

[34] Osteryoung K.W., Pyke K.A. (2014). Division and dynamic morphology of plastids. Annu. Rev. Plant Biol..

[35] Vitha S., McAndrew R.S., Osteryoung K.W. (2001). FtsZ ring formation at the chloroplast division site in plants. J. Cell Biol..

[36] Osteryoung K.W., Stokes K.D., Rutherford S.M., Percival A.L., Li W.Y. (1998). Chloroplast division in higher plants requires members of two functionally divergent gene families with homology to bacterial ftsZ. Plant Cell.

[37] Strepp R., Scholz S., Kruse S., Speth V., Reski R. (1998). Plant nuclear gene knockout reveals a role in plastid division for the homolog of the bacterial cell division protein FtsZ, an ancestral tubulin. Proc. Natl. Acad. Sci. USA.

[38] Donachie W.D. (1993). The cell cycle of *Escherichia coli*. Annu. Rev. Microbiol..

[39] Margolin W. (2005). FtsZ and the division of prokaryotic cells and organelles. Nat. Rev. Mol. Cell Biol..

[40] TerBush A.D., Yoshida Y., Osteryoung K.W. (2013). FtsZ in chloroplast division: Structure, function and evolution. Curr. Opin. Cell Biol..

[41] Yoshida Y., Mogi Y., TerBush A.D., Osteryoung K.W. (2016). Chloroplast FtsZ assembles into a contractible ring via tubulin-like heteropolymerization. Nat. Plants.

[42] Lutkenhaus J.F., Wolf-Watz H., Donachie W.D. (1980). Organization of genes in the ftsA-envA region of the *Escherichia coli* genetic map and identification of a new fts locus (ftsZ). J. Bacteriol..

[43] Schmitz A.J., Glynn J.M., Olson B.J., Stokes K.D., Osteryoung K.W. (2009). *Arabidopsis* FtsZ2-1 and FtsZ2-2 are functionally redundant, but FtsZ-based plastid division is not essential for chloroplast partitioning or plant growth and development. Mol. Plant.

[44] McAndrew R.S., Froehlich J.E., Vitha S., Stokes K.D., Osteryoung K.W. (2001). Colocalization of plastid division proteins in the chloroplast stromal compartment establishes a new functional relationship between FtsZ1 and FtsZ2 in higher plants. Plant Physiol..

[45] Stokes K.D., McAndrew R.S., Figueroa R., Vitha S., Osteryoung K.W. (2000). Chloroplast division and morphology are differentially affected by overexpression of FtsZ1 and FtsZ2 genes in Arabidopsis. Plant Physiol..

[46] de Pater S., Caspers M., Kottenhagen M., Meima H., ter Stege R., de Vetten N. (2006). Manipulation of starch granule size distribution in potato tubers by modulation of plastid division. Plant Biotechnol. J..

[47] Yun M.S., Kawagoe Y. (2010). Septum formation in amyloplasts produces compound granules in the rice endosperm and is regulated by plastid division proteins. Plant Cell Physiol..

[48] Occhialini A., Pfotenhauer A.C., Frazier T.P., Li L., Harbison S.A., Lail A.J., Mebane Z., Piatek A.A., Rigoulot S.B., Daniell H. (2020). Generation, analysis, and transformation of macro-chloroplast potato (*Solanum tuberosum*) lines for chloroplast biotechnology. Sci. Rep..

[49] Christensen D.H., Madsen M.H. (1996). Changes in potato starch quality during growth. Potato Res..

[50] Li M., Li X., Zhou Z., Wu P., Fang M., Pan X., Lin Q., Luo W., Wu G., Li H. (2016). Reassessment of the four yield-related genes *Gn1a, DEP1, GS3,* and *IPA1* in rice using a CRISPR/Cas9 system. Front. Plant Sci..

[51] Zhu S., Yu X., Li Y., Sun Y., Zhu Q., Sun J. (2018). Highly efficient targeted gene editing in upland cotton using the CRISPR/Cas9 system. Int. J. Mol. Sci..

[52] Bánfalvi Z., Csákvári E., Villányi V., Kondrák M. (2020). Generation of transgene-free *PDS* mutants in potato by *Agrobacterium*-mediated transformation. BMC Biotechnol..

[53] Stupar R.M., Lilly J.W., Town C.D., Cheng Z., Kaul S., Buell C.R., Jiang J. (2001). Complex mtDNA constitutes an approximate 620-kb insertion on *Arabidopsis thaliana* chromosome 2: Implication of potential sequencing errors caused by large-unit repeats. Proc. Natl. Acad. Sci. USA.

[54] Lough A.N., Roark L.M., Kato A., Ream T.S., Lamb J.C., Birchler J.A., Newton K.J. (2008). Mitochondrial DNA transfer to the nucleus generates extensive insertion site variation in maize. Genetics.

[55] Michalovova M., Vyskot B., Kejnovsky E. (2013). Analysis of plastid and mitochondrial DNA insertions in the nucleus (NUPTs and NUMTs) of six plant species: Size, relative age and chromosomal localization. Heredity.

[56] Puertas M.J., González-Sánchez M. (2020). Insertions of mitochondrial DNA into the nucleus-effects and role in cell evolution. Genome.

[57] McAndrew R.S., Olson B.J., Kadirjan-Kalbach D.K., Chi-Ham C.L., Vitha S., Froehlich J.E., Osteryoung K.W. (2008). In vivo quantitative relationship between plastid division proteins FtsZ1 and FtsZ2 and identification of ARC6 and ARC3 in a native FtsZ complex. Biochem. J..

[58] Okechukwu P.E., Rao M.A. (1995). Influence of granule size on viscosity of cornstarch suspension. J. Texture Stud..

[59] Rao M.A., Tattiyakul J. (1999). Granule size and rheological behavior of heated tapioca starch dispersions. Carbohydr. Polym..

[60] Kumar R., Khatkar B.S. (2017). Thermal, pasting and morphological properties of starch granules of wheat (*Triticum aestivum* L.) varieties. J. Food Sci. Technol..

[61] Waterschoot J., Gomand S.V., Fierens E., Delcour J.A. (2014). Starch blends and their physicochemical properties. Starch.

[62] Zhu F., Hua Y., Li G. (2020). Physicochemical properties of potato, sweet potato and quinoa starch blends. Food Hydrocoll..

[63] Waterschoot J., Gomand S.V., Willebrords J.K., Fierens E., Delcour J.A. (2014). Pasting properties of blends of potato, rice and maize starches. Food Hydrocoll..

[64] Chung K.M., Moon T.W., Kim H., Chun J.K. (2002). Physicochemical properties of sonicated mung bean, potato, and rice starches. Cereal Chem..

[65] Scott S.E., Inbar Y., Wirz C.D., Brossard D., Rozin P. (2018). An overview of attitudes toward genetically engineered food. Annu. Rev. Nutr..

[66] Lusk J.L., McFadden B.R., Wilson N. (2018). Do consumers care how a genetically engineered food was created or who created it?. Food Policy.

[67] Menz J., Modrzejewski D., Hartung F., Wilhelm R., Sprink T. (2020). Genome edited crops touch the market: A view on the global development and regulatory environment. Front. Plant Sci..

[68] Kim S., Kim D., Cho S.W., Kim J., Kim J.S. (2014). Highly efficient RNA-guided genome editing in human cells via delivery of purified Cas9 ribonucleoproteins. Genome Res..

[69] Zhang Y., Liang Z., Zong Y., Wang Y., Liu J., Chen K., Qiu J.L., Gao C. (2016). Efficient and transgene-free genome editing in wheat through transient expression of CRISPR/Cas9 DNA or RNA. Nat. Commun..

[70] Si X., Zhang H., Wang Y., Chen K., Gao C. (2020). Manipulating gene translation in plants by CRISPR-Cas9-mediated genome editing of upstream open reading frames. Nat. Protoc..

[71] Xing H.L., Dong L., Wang Z.P., Zhang H.Y., Han C.Y., Liu B., Wang X.C., Chen Q.J. (2014). A CRISPR/Cas9 toolkit for multiplex genome editing in plants. BMC Plant Biol..

[72] Curtis M.D., Grossniklaus U. (2003). A gateway cloning vector set for high-throughput functional analysis of genes *in planta*. Plant Physiol..

[73] Haeussler M., Schonig K., Eckert H., Eschstruth A., Mianne J., Renaud J.B., Schneider-Maunoury S., Shkumatava A., Teboul L., Kent J. (2016). Evaluation of off-target and on-target scoring algorithms and integration into the guide RNA selection tool CRISPOR. Genome Biol..

[74] Weigel D., Glazebrook J. (2006). Transformation of *Agrobacterium* using the freeze-thaw method. CSH Protoc..

[75] Chronis D., Chen S., Lang P., Tran T., Thurston D., Wang X. (2014). Potato transformation. Bio-Protocol.

[76] Lin M.T., Occhialini A., Andralojc P.J., Parry M.A.J., Hanson M.R. (2014). A faster rubisco with potential to increase photosynthesis in crops. Nature.

[77] Liu Q., Weber E., Currie V., Yada R. (2003). Physicochemical properties of starches during potato growth. Carbohydr. Polym..

[78] Mendez-Montealvo G., Wang Y.J., Campbell M. (2011). Thermal and rheological properties of granular waxy maize mutant starches after β-amylase modification. Carbohydr. Polym..

