# Supplementary material for: Genome-Editing of FtsZ1 for Alteration of Starch Granule Size in Potato Tubers
Source: Plants (Basel). 2023 May 4;12(9):1878. doi: 10.3390/plants12091878 (PMC10180631; doi:10.3390/plants12091878)
Supplement: Supplementary file 1 [file plants-12-01878-s001.zip › plants-2301198-supplementary.pdf]

Supplementary Table S1. *Mitochondrial DNA insertion in line 3.16*. The 412 and 777 base pair insertion in line 3.16 aligns with 100% homology to S. tuberosum var. 'Desirée' BLAST sequence ID: MN104801.1.

**Line 3.16 mitochondrial DNA insertion, BLAST ID: MN104801.1.**
**412 basepair insertion**
CTCTACCGACAGATGCGCGAATTGCACTGGTCATTGTGCGGAAT<br>GCCCTCTAAGCCAGTCAGTCAATGGAGGGACCGGGGCGCTAG<br>GTATTTTCTTTCTTCTGGCCGTAGGTGTGATCAATGCCATTGAG<br>CTTCGGTACTCTAAAATAGTAGTCGGAATTCGCTTCTGAGTGAG<br>CTTCCTTCCTTATGCTCTGGCCTGACCTTCACGAACAGCTTTCTT<br>TCAGCTACCAACTTCAAAGCTTGATCTCATCTCTCTTCTTTTGAAA<br>GTTTAGTACTATTATTGTTGGCATGGAAGGAGTTCATCTTGAATT<br>GCCAGTACAGTGATTGGAGGCCTTCCTCACCTGTTCTAGTGACA<br>TAGGCCCATCCGGTCGAGAGAAGAGACTTGAGATTAGCCACTA<br>GGTGAAGTACCAAGG
**777 basepair insertion**
 AGAAGAAAGTATTCTGAAGCCAAATGCTCTTGGATGGGTAGTTG<br>AATAGGGGGATAAATCCTTTTTTCTCCTTTATATTGAATGTAGTC<br>AATCATTTTGAGGTCATGAGAACAAAGAGTCATAGAGATGTGAG<br>AAGTTTTGCTTTCGCTATCGACTGACCGTCCTGTTAGCAGTGCT<br>ATACTAGATTGGCACCCTAACTGGTACCTATACAGCTACTCCTTG<br>TCAGTTGCTATATTTTTTCACCTTGAGGCTGAGAGGTTAACTCCT<br>TCCACTCTCAACCGCCTTTGCTTATCTTACTAGTAGCTCTATCTAT<br>TGGTACCAGCCAGGGAACTCCCTCTTTGCGAGCTACTTACTTTA<br>TCTGAACCCTCTACCGACAGATGCGCGAATTGCACTGGTCATTG<br>TGCGGAATGCCCTCTAAGCCAGTCAGTCAATGGAGGGACCGGG<br>GCGCTAGGTATTTTCTTTCTTCTGGCCGTAGGTGTGATCAATGC<br>CATTGAGCTTCGGTACTCTAAAATAGTAGTCGGAATTCGCTTCT<br>GAGTGAGCTTCCTTCCTTATGCTCTGGCCTGACCTTCACGAACA<br>GCTTTCTTTCAGCTACCAACTTCAAAGCTTGATCTCATCTCTCTTC<br>TTTTGAAAGTTTAGTACTATTATTGTTGGCATGGAAGGAGTTCAT<br>CTTGAATTGCCAGTACAGTGATTGGAGGCCTTCCTCACCTGTTC<br>TAGTGACATAGGCCCATCCGGTCGAGAGAAGAGACTTGAGATT<br>AGCCACTAGGTGAAGTACCAAGG

Supplementary Table S2. *Microtuber weight and mean area.* Lines of similar weight were chosen in order to avoid the possibility that larger tubers would contain larger starch granules. Weight, mean area, standard deviation, and number of granules measured by ImageJ 1.41 from the National Institute of Health are shown. WT = wild-type.

Line	Tuber weight (mg)	Granule mean area (μm^2)	Standard deviation	n
1.2	10.3	157.38	135.53	1740
1.3	19.7	162.68	150.10	1428
1.5	10.6	151.70	134.24	1943
2.6	10.4	123.83	91.77	4480
2.7	17.5	116.37	76.24	3147
2.9	15.3	107.47	71.55	8150
3.3	23.6	197.56	167.06	1069
3.8	10	175.44	179.68	461
3.9	24.6	284.90	171.97	765
3.12	12.1	157.56	128.40	2879
3.13	21	159.43	123.31	2172
3.16	28.4	148.80	131.19	733
3.18	11.5	159.51	141.12	707
4.1	25.5	165.38	136.89	1175
4.2	10.5	110.27	97.58	1529
4.3	11.2	166.59	127.23	827
WT-A	13.6	134.33	100.28	4062
WT-B	18.2	137.92	108.53	1805
WT-C	29.7	143.95	120.28	861

Supplementary Table S3. Primers and gRNAs. Primers and gRNAs used in this study are shown. The top number in the gRNA off-target cell refers to the number of off-targets per number of mismatches. For example, 0-12-24-36-48 means 0 off-targets with 0 mismatches, 12 off-targets with 1 mismatch, 24 off-targets with 2 mismatches, etc. The bottom number in the off-target cell refers to the off-targets with mismatches in the 12 bp flanking the PAM sequence, which are the most likely off-targets. Restriction enzymes targeted by the gRNAs for use in the restriction enzyme digest assay are shown. gRNA = guide RNA. PAM = protospacer adjacent motif.

**Primers used 5' >> 3'**				
Name		Purpose		
1	AATATTAAGCTTATGGCGATTTTAGGGCTCTCA	Clone FtsZ cDNA		
2	GTATGCTCTAGACTAAAAGAACAGCCTTCGAGT	Clone FtsZ cDNA		
3	CAGTGGCCATGGATGAAAAAGCCTGAACTCACC	Clone hyg cassette		
4	CGATTTTGAAACCGCGGTGA	Clone hyg cassette		
5	CCCTGAAGTCAAAGCTCGTC	Check plants for Cas9		
6	GGCGATCATCTTCCTCACAT	Check plants for Cas9		
7	GGATTTTCTATCAGTTGGCT	gRNA 1, 2 RED		
8	AAGAGGATTGCCACCAGTAC	gRNA 1, 2 RED		
9	CTTCTGACTCGTGGGCTTGG	gRNA 3, 4, 5 RED		
10	CCTGCACAGATCTTTTACGTCC	gRNA 3, 4, 5 RED		
11	ATGGCGATTTTAGGGCTCTC	FtsZ CDS RT-PCR		
12	CTAAAAGAACAGCCTTCGAGTAGG	FtsZ CDS RT-PCR		
13	ACAGTCTGCTGCCGAGA	FtsZ qRT-PCR		
14	CTCCGCTGCCTGTTCCC	FtsZ qRT-PCR		
15	GATGGTCAGACACGTGAACA	EF1α qRT-PCR		
16	CCTTGGAGTACTTGGGGGTG	EF1α qRT-PCR		
**gRNAs used 5' >> 3'**				
Name	Sequence	GC content	Off-target effect	Corresponding restriction enzyme
gRNA1-Fw	attgAAACACGGATGCTCAAGCAC	50	0-0-0-1-12	BtsIMutI
gRNA1-Rv	aaacGTGCTTGAGCATCCGTGTTT		0-0-0-0-0	
gRNA2-Fw	attgATTGGAGAACTTCTGACTCG	45	0-0-1-0-17	BssSaI
gRNA2-Rv	aaacCGAGTCAGAAGTTCTCCAAT		0-0-0-0-0	
gRNA3-Fw	attgGCTGCCTGTTCCCCTAAAAG	55	0-0-0-5-10	EcoNI
gRNA3-Rv	aaacCTTTTAGGGGAACAGGCAGC		0-0-0-0-0	
gRNA4-Fw	attgTTTAGGGGAACAGGCAGCGG	60	0-0-0-0-7	MspA1I
gRNA4-Rv	aaacCCGCTGCCTGTTCCCCTAAA		0-0-0-0-0	
gRNA5-Fw	attgGCAGGAATGGGTGGAGGTAC	60	0-0-2-5-36	RsaI
gRNA5-Rv	aaacGTACCTCCACCCATTCCTGC		0-0-0-0-1	

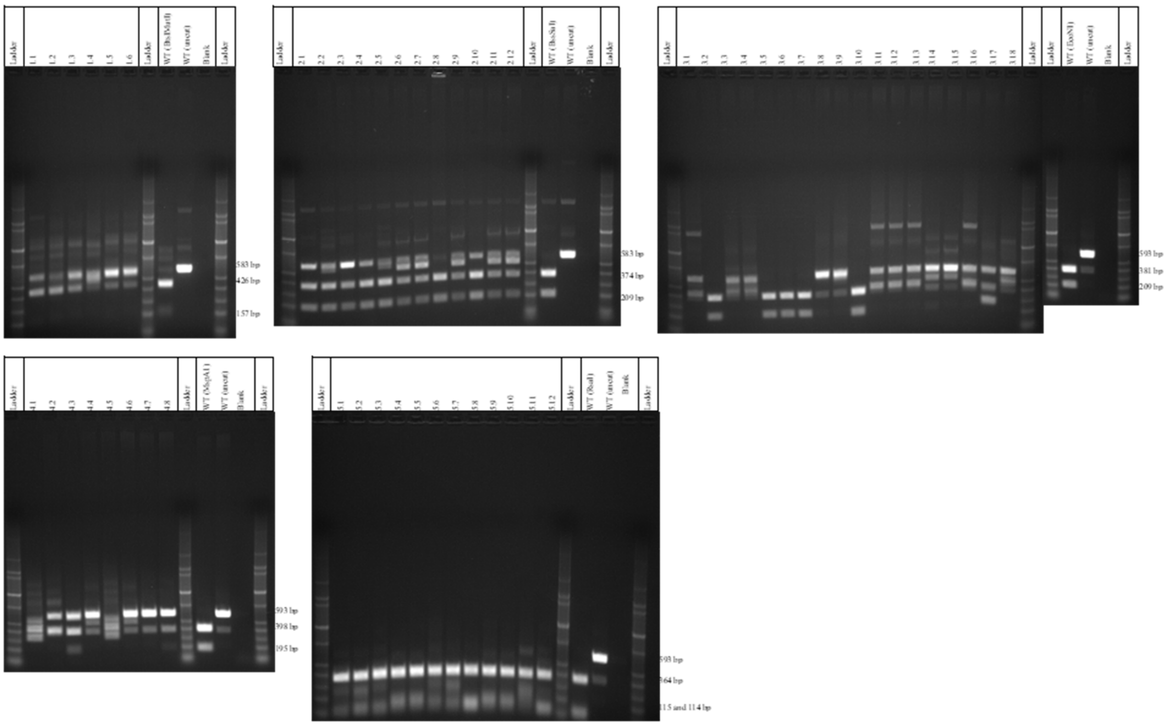

Supplementary Figure S1. *Restriction digest assay.* Genomic DNA from 56 putative transgenic plants was extracted and PCR amplified. Fragments were then digested with appropriate restriction enzymes overnight and ran on an agarose gel. A higher molecular weight band is indicative of Cas9 activity, subsequent DNA repair, and loss of a restriction enzyme recognition site. WT = wild-type.

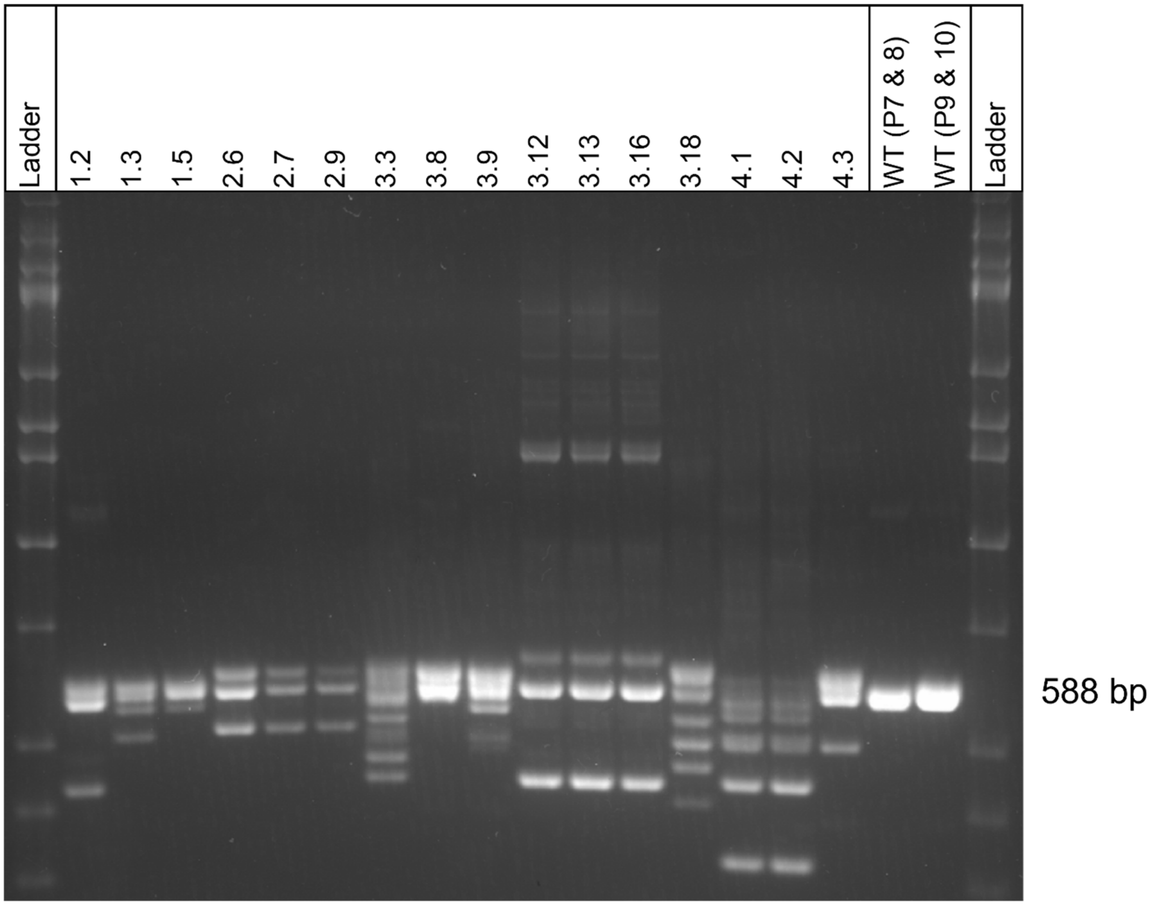

Supplementary Figure S2. *PCR amplification of FtsZ1 fragments.* PCR was conducted on the 16 down-selected lines with primers 7 & 8 or 9 & 10. Lines 1.2 – 2.9 were amplified with primers 7 & 8. Lines 3.3 – 4.3 were amplified with primers 9 & 10. WT = wild-type. P = primer.

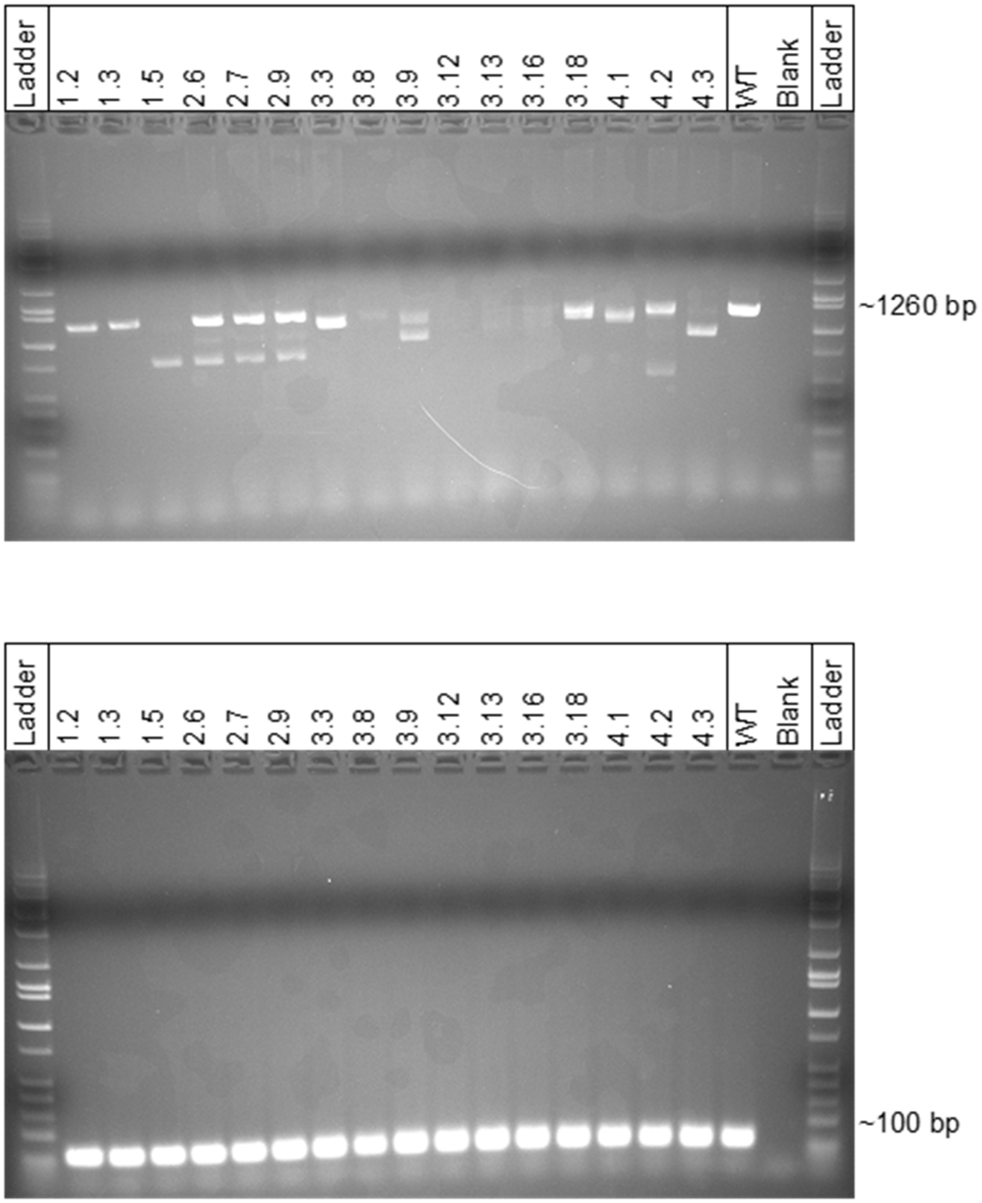

Supplementary Figure S3. FtsZ1 *expression patterns.* RT-PCR was conducted on full length 1260 bp coding sequence of FtsZ1 (A). RT-PCR was conducted on 100 bp fragment of EF1α as a control (B). WT = wild-type.

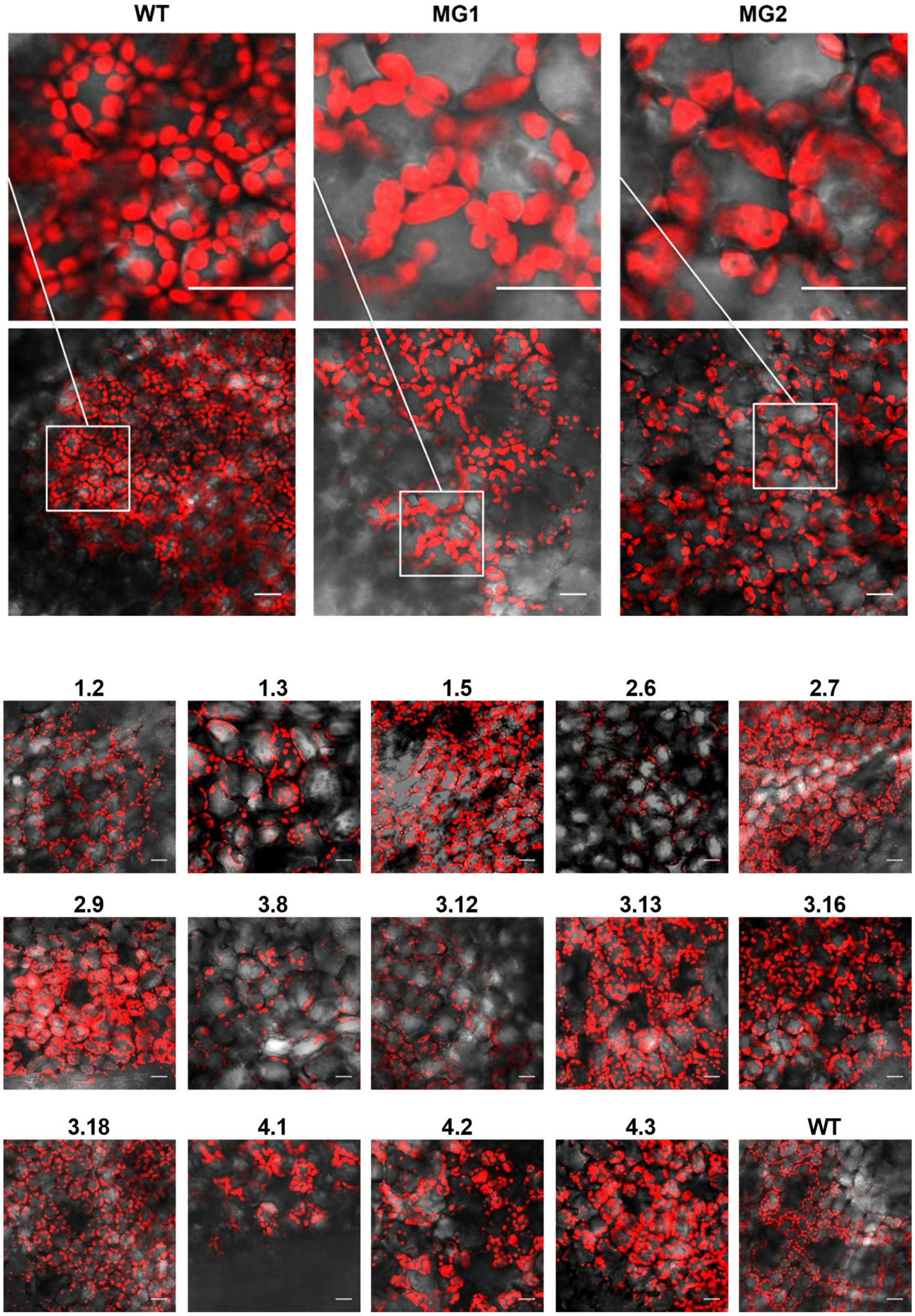

Supplementary Figure S4. *Chloroplast morphology by confocal microscopy of large starch granule lines.* Leaf tissue from mutants and wild-type were screened for chloroplast abnormalities. WT = wild-type. MG1 = MacroGranule1. MG2 = MacroGranule2. Scale bar = 25 μm.

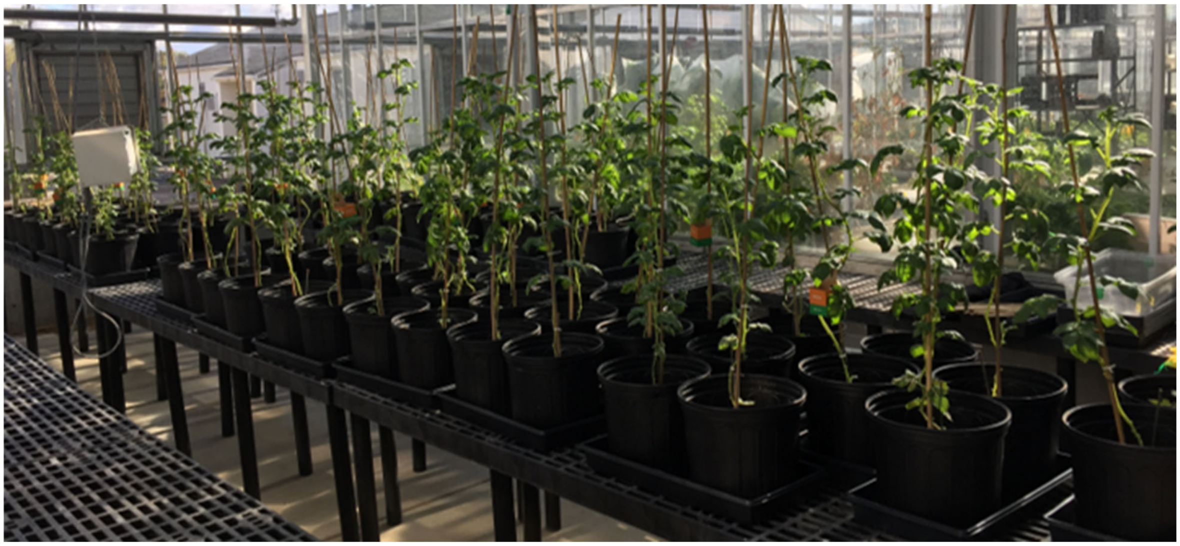

Supplementary Figure S5. *Greenhouse production of FtsZ1 mutant potato lines.* Plants were grown in tubers until senescence for the production of tubers.

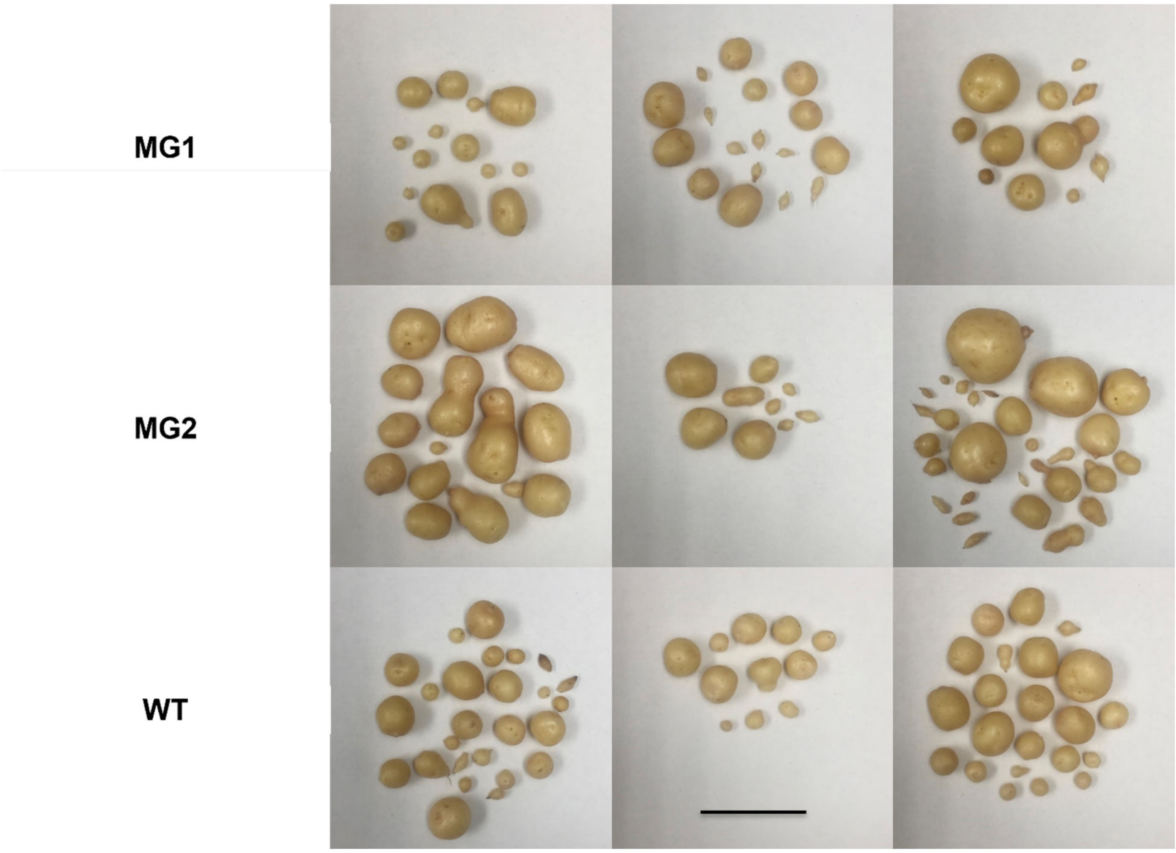

Supplementary Figure S6. *Tubers produced from MacroGranule1, MacroGranule2, and WT.* Plants were grown in a greenhouse until senescence to produce tubers. Tubers from three plant replicates were produced and are shown in the different panels. Scale bar = 5 cm. WT = wild-type. MG1 = MacroGranule1. MG2 = MacroGranule2.
